# Prevalence of HBV and HCV among blood donors in Kosovo

**DOI:** 10.1186/1743-422X-6-21

**Published:** 2009-02-13

**Authors:** Hajrullah Fejza, Skender Telaku

**Affiliations:** 1 Sector for Public Health, Municipality of Prishtina, Kosovo; 2 Gastroenterology Unit, University Clinical Center of Kosovo, Prishtina, Kosovo

## Abstract

Hepatitis is disease of the liver caused by the infectious and non-infectious agents.

The aim of study was to analyze the prevalence of HBV and HCV among voluntary blood donors in Kosovo, during 2000–2003.

The data from National Center for Blood Transfusion of Kosovo were collected and analyzed through descriptive and comparative epidemiological method of retrospective study. All samples were tested by ELISA test.

Out of 70348 samples of the blood donors, 3145 were positive. From overall positive samples, 2939 were HBV positive, 192 HCV positive while 14 samples were positive for both viruses.

The HBV prevalence among the blood donors of Kosovo is 4.2%, which range Kosovo to the second zone according to the CDC classification of the geographical spread of the HBV infection.

The HCV prevalence among the blood donors in Kosovo is 0.3%. Compared to the other European countries this level of prevalence is relatively low.

Age group 30–39 years old was presented with 34.8% of cases. The higher number was among the workers, 842 or 26.8%.

Based on the results we can conclude that Kosovo have the similar prevalence for HBV and HCV infections as other South East European countries.

## Introduction

Hepatitis is term to describe a nonspecific liver inflammation [1-5]. Until now are known 8 types of hepatitis: A, B, C, D, E, F, G and TT. Hepatitis B and C are similar types of liver infection, which are mostly spread through blood and blood products.

The possibility of hepatitis transmission through blood and blood products were known since 1950 [6-9].

Hepatitis B virus is an AND virus from hepadnaviridae family. Hepatitis C virus is an ARN virus with lipid coat similar to flaviviridae family.

Infected person or asymptomatic carriers with viral hepatitis B and C are only reservoir of infection [8-12].

Researches show as that world prevalence of HBsAg carriers is from 0.1% till 20% with high percentage in tropical countries [5,12].

The prevalence of anti HCV antibody is variety in different world countries with high number reported for Egypt.

## Aim of study

The aim of a study was to analyze the prevalence of the HBsAg and anti-HCV antibodies in Kosovo during the period 2000–2003. The possible influence of the various factors on the prevalence was analyzed too. The prevalence was compared with the data available on European and World level.

## Material and methodology

Data from databank of the voluntary blood donors of the National Blood Bank in Pristina, as well the data from the databank of the Transfusion Centers in regional Hospitals in Peja, Gjakova, Prizren and Gjilan were used in this study. The analyzed period was from 2000 – 2003.

The method of study was descriptive and comparative in retrospective aspect.

The T – test and Χ^2^- test were used to analyze the significance of the results.

All samples were tested by ELISA test.

## Results

**Figure 1:**
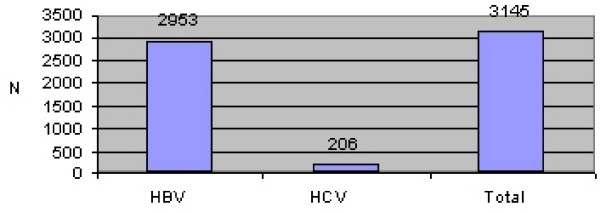
**Infected persons with Hepatitis B and C viruses, Kosovo 2000–2003**.

The results of the study showed that from 70348 samples of the blood donated by the blood donors, 3145 were positive. From overall positive samples, 2939 were HBV positive, 192 HCV positive, while in 14 samples both viruses were discovered (Table 1 and Figure 1)

**Table 1:** Prevalence of HBV and HCV, Kosovo 2000–2003, by type.

Type of Infection	HBV		HCV		Total	
	
	N	%	N	%	N	%
Mono-infection	2939	99.5	192	93.2	**3131**	**99.6**

Bi-infection	14	0.5	14	6.8	**14**	**0.4**

Total	2953	100.0	206	100.0	3145	100.0

**Figure 2:**
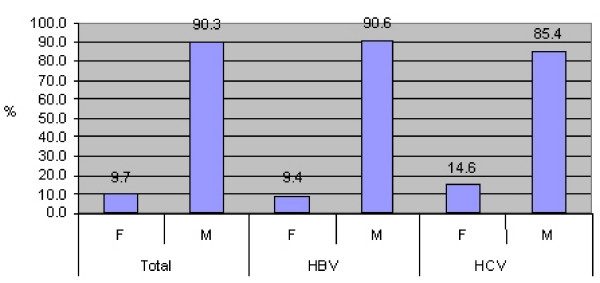
**Infection with HBV and HCV among blood donors in Kosovo, 2000–2003 By type and sex**.

Male sex is represented with higher number of infected persons with HBV and HCV. From overall infected persons, 90.3% are male and only 9.7% are female. The same values are shown in infection with HBV whereas a little grow is shown in infection with HCV where female sex is represented with 14.6% (Figure 2)

The most of infected persons with HBV and HCV belongs to age group from 20–29 years and from 30–39 years.

Higher number is registered in age group from 30–39 years with value of 34.8%, whereas age group from 20–29 years is represented with 33.3%. The less value is shown in age group over 50 year with 4.9%. The average of infected persons is 32.2 year with SD 9.4 (Table 2)

**Table 2:** Infection with HBV and HCV among blood donors in Kosovo, by age group and years.

	Year				Total	
Age group	2000	2001	2002	2003	N	%

10–19	32	52	126	77	287	9.1

20–29	260	256	322	209	1047	33.3

30–39	262	294	308	232	**1096**	**34.8**

40–49	137	113	170	140	560	17.8

50+	44	25	59	27	155	4.9

Total	735	740	985	685	3145	100.0

Average	33.3	32.0	31.7	32.0	**32.2**	-

SD	9.3	8.8	10.0	9.4	**9.4**	-

**Figure 3:**
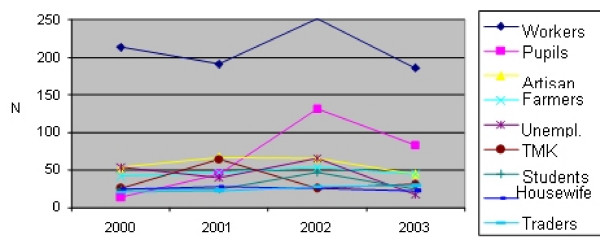
**Infection with HBV and HCV among blood donors in Kosovo, 2000–2003 By occupation and years**.

The blood donors which samples were analyzed belong to different occupations. However, the highest number, 842 (26.8%) were workers, followed by pupils with 7.3%. The lowest number was among traders, 3.1%, (Figure 3).

## Discussion

Infection with HBV and HCV are worldwide significant problem in public health [13-18]. About 5% (300 millions), of world population has chronic infection HBV, which is major factor for developing of chronic liver cirrhosis and carcinoma hepatocelulare [19-23].

According to CDC estimation about 3.9 million people worldwide are infected with HCV, with highest prevalence among age group 30–39 year and about 8000–10 000 death/year from lives disease caused from HCV infection, (CDC, 1998).

The prevalence of HCV in world level can be more than 3%, [1,24-27].

Out of 70348 samples of the blood donors, 3145 were positive. From overall positive samples, 2939 were HBV positive, 192 HCV positive while 14 samples were positive for both viruses.

The HBV prevalence among the blood donors of Kosovo is 4.2%, which range Kosovo to the second zone according to the CDC classification of the geographical spread of the HBV infection.

According to the study done in 1992 among blood donors in Kosovo, the prevalence of HBV was 12, 05%, [21]. We can conclude that after the adequate preventives measures in this period, the prevalence of HBV is decreased significantly.

The prevalence of HBV in dialyses patients in Kosovo is 24.2%, [28]. We think that such highest prevalence is caused because of continually percutane blood exposure.

The HCV prevalence among the blood donors in Kosovo is 0.3%. Compared to the other European countries this level of prevalence is relatively low.

According to the WHO, the world prevalence with HCV is 3.1% [29,30]. The highest prevalence is in Africa, 5.3%, whereas the lowest prevalence is in Europe, 1.03%, [30]. The highest prevalence of HCV between countries in whole the world is in Egypt, 6–28% (mean 22%), [19,31-33].

We can conclude that lower prevalence of HCV in Kosovo is because here are not a lot of people living in high-risk groups for infection with HCV.

The prevalence of HCV among dialyses patients in Kosovo is 38.8%, [28]. This finding can confirm the meaning that long exposure to blood and blood products is with high risk for infection with HCV.

Age group 30–39 years old was presented with 34.8% of cases. The higher number was among the workers, 842 or 26.8%.

## Conclusion

• Prevalence of HBV among blood donors in Kosovo is 4.2%.

• Prevalence of HCV among blood donors in Kosovo is 0.3%.

• Based on the results we can conclude that we have the similar prevalence for HBV and HCV infections as other southeast European countries.

## Proposition of measures

• To ensure health education activities among population regarding those infections,

• To effort programs and projects which mean the activities with risk group population insisting of them to use condoms and another protection measures during sexual activities and professional care.

• To find sources for completing the lab with best tests such are RIBA and PCR.

## Competing interests

The authors declare that they have no competing interests.

## Authors' contributions

HF carried about data collecting, participated in design of the study and drafted manuscript. ST participated in design of the study and performed the statistical analysis. Both authors read and approved the final manuscript.
